# Study on the Microbial Inactivation and Quality Assurance of Ultrasonic-Assisted Slightly Acidic Electrolyzed Water for Mirror Carp (*Cyprinus carpio* L.) Fillets During Refrigerated Storage

**DOI:** 10.3390/foods14152652

**Published:** 2025-07-29

**Authors:** Qiang Zhong, Xiufang Xia, Fangfei Li

**Affiliations:** 1 College of Food Science, Northeast Agricultural University, Harbin 150030, China; zq887721@163.com; 2 College of Life Science, Northeast Forestry University, Harbin 150030, China

**Keywords:** ultrasound, slightly acidic electrolyzed water, microbial inactivation, aquatic product preservation, refrigerated storage

## Abstract

The advancement of non-thermal disinfection technologies represents a critical pathway for ensuring food safety, meeting environmental sustainability requirements, and meeting consumer preferences for clean-label products. This study systematically evaluated the combined preservation effect of ultrasonic-assisted slightly acidic electrolyzed water (US+SAEW) on mirror carp fillets during refrigeration. Results demonstrated that US+SAEW exhibited superior antimicrobial efficacy compared to individual US or SAEW, achieving reductions of 0.73, 0.74, and 0.79 log CFU/g in total viable counts (TVC), *Aeromonas* bacteria, and lactic acid bacteria counts compared to the control, respectively. Furthermore, the combined intervention significantly suppressed microbial proliferation throughout the refrigeration period while simultaneously delaying protein and lipid degradation/oxidation induced by spoilage bacteria, thereby inhibiting the formation of alkaline nitrogenous compounds. Consequently, lower levels of pH, total volatile basic nitrogen (TVB-N), protein carbonyl, and thiobarbituric acid reactive substances (TBARS) were observed in US+SAEW compared to the other treatments. Multimodal characterization through low-field nuclear magnetic resonance (LF-NMR), texture, and color analysis confirmed that US+SAEW effectively preserved quality characteristics, extending the shelf life of mirror carp fillets by four days. This study provides a novel non-thermal preservation strategy that combines microbial safety maintenance with quality retention, offering particular advantages for thermolabile food.

## 1. Introduction

With the advancement of cold-chain logistics and the prepared food industry, refrigerated freshwater fish fillets have emerged as a convenient and essential ingredient for the catering field. Among these, mirror carp (*Cyprinus carpio* L.) fillet stands out for its economic significance, palatability, and nutritional value [1]. Nevertheless, the high moisture and protein content of mirror carp fillets render them susceptible to microbial spoilage during storage, leading to quality degradation through water loss, textural softening, and off-taste development [2]. These challenges necessitate innovative preservation strategies to inhibit microbial proliferation while maintaining product quality. Growing consumer demand for environmentally sustainable and health-conscious food processing has accelerated the adoption of non-thermal disinfection technologies. Such methods are particularly suitable for heat-sensitive foods such as fish fillets, where conventional thermal treatments may compromise sensory attributes. However, single non-thermal approaches often suffer from inefficiency and excessive energy consumption [3]. Emerging evidence suggests that combined multi-technology strategies can enhance disinfection efficacy, reduce processing duration, and better preserve food quality in aquatic products [4,5].

Ultrasonic (US) disinfection leverages cavitation effects to disrupt microbial cell integrity through mechanical stress and reactive oxygen species generation [6], leading to the leakage of bacterial contents and facilitating the entry of external substances into the cells [7]. This non-thermal method offers several advantages, including broad-spectrum efficacy, strong penetrability, and suitability for heat-sensitive products. Importantly, this method complies with sustainability and environmental standards due to its eco-friendly processing characteristics [8]. However, single US exhibits limited disinfection efficiency, while high power and prolonged treatment increase energy consumption and may induce food oxidation [9,10], consequently compromising quality attributes. These limitations can be effectively addressed through combining with other technologies [11,12].

Slightly acidic electrolyzed water (SAEW) can be prepared via electrolysis of sodium chloride (NaCl) solution and dilute hydrochloric acid (HCl), which has been regarded as a promising disinfectant for food products [13]. As the main functional/antibacterial ingredient of SAEW, hypochlorous acid (HClO) can oxidize microbial intracellular enzymes and destroy the structure of enzymes [14]. Importantly, SAEW is produced from safe raw materials, demonstrating both cost-effectiveness and excellent biosafety profiles. Meanwhile, SAEW is shown to be environmentally friendly because it can turn into ordinary water without harmful residues upon dilution [15]. However, achieving effective disinfection with SAEW alone requires prolonged exposure or elevated available chlorine concentrations, which may adversely affect food sensory properties [16]. Therefore, combining SAEW with other disinfection methods is an effective strategy to overcome these difficulties.

The combination of US and SAEW is a safe and effective disinfection strategy with promising potential for industrial-scale applications. Currently, the combined application of US and SAEW has primarily focused on controlling foodborne pathogens [7,12,17], preserving agricultural products [18,19], and seafood decontamination [20]. However, its application in freshwater fish remains scarce. Notably, the dominant spoilage bacteria in freshwater fish during storage might differ from those in other products, necessitating investigation into the efficacy of combined US and SAEW treatment in eliminating specific spoilage microorganisms in freshwater fish, as well as evaluating its feasibility for industrial-scale preservation. Moreover, since the basic composition (e.g., lipids, proteins) of freshwater fish may also differ from other products, applying appropriate parameters of US and SAEW to prevent quality deterioration is crucial. Therefore, this study systematically evaluates the antibacterial effects of combined US and SAEW treatment against *Aeromonas* and lactic acid bacteria in refrigerated mirror carp fillets while also assessing its effects on oxidative stability and overall quality during refrigerated storage. The findings will provide a theoretical foundation and practical parameter references for the industrial application of US combined with SAEW in freshwater fish preservation.

## 2. Materials and Methods

### 2.1. Sample and Material Preparation

Mirror carp were purchased from a local supermarket (Harbin, China). The fish were anesthetized in a benzocaine solution (0.4%) for 20 min and killed by percussive stunning under the condition of minimizing stress performed by the trained staff. Subsequently, they were beheaded, eviscerated, and transported to the laboratory. All procedures complied with the guidelines for the treatment of experimental animals (2006) issued by the Ministry of Science and Technology of China and were approved by the Animal Care Committee from Northeast Agricultural University (Harbin, China). The mirror carp were cut into fillets (30 ± 2 g) and stored in zipper bags at 4 °C for later use. The parameters of the ultrasonic bath system (Nanjing Xian ou Co., Ltd., Nanjing, China) were set to a power of 200 W, frequency of 30 kHz, and a temperature of 4 °C. SAEW (pH 6.2, 60 mg/L available chlorine) was prepared by electrolyzing a solution containing 5% NaCl and 1% HCl using an SAEW generator (Yantai Fang Xin Water Treatment Equipment Co., Ltd., Yantai, China).

### 2.2. US-Assisted SAEW Treatment

Mirror carp fillets were randomly allocated to four treatment conditions: (1) Control (no treatment); (2) US (US treatment for 5 min); (3) SAEW (immersion in SAEW (1:2, m/v) for 5 min); and (4) US+SAEW (combined SAEW immersion (1:2, m/v) with simultaneous US treatment for 5 min). After treatment, all samples were drained, packaged in zipper bags, and stored at 4 °C for 10 days.

### 2.3. Microbiological Characterization

Mirror carp fillet (10 g) was immersed in sterilized chilled saline water (90 mL) and homogenized at 8000 r/min for 1 min. Appropriate dilution (1 mL) was spread on the plate count agar, *Aeromonas* Medium Base (RYAN), and Man Rogosa Sharpe agar to analyze the total viable count (TVC), *Aeromonas* counts, and lactic acid bacteria counts, respectively. The plate was cultured in a 37 °C incubator for 48 ± 2 h, and the bacterial counts were recorded.

### 2.4. pH

Mirror carp fillet (5.0 g) was homogenized with 45 mL distilled water at 8000 r/min for 1 min. After filtering, the filtrate pH was measured using a calibrated pH meter (Mettler Toledo Instrument (Shanghai) Co., Ltd., Shanghai, China). Each test was performed three times.

### 2.5. Total Volatile Basic Nitrogen (TVB-N)

TVB-N was performed using the Kjeldahl method as previously described [21], and expressed as mg/100 g of mirror carp sample.

### 2.6. Protein and Lipid Oxidation

Protein carbonyl content determination was fully performed as described by Zhang et al. [22]. Thiobarbituric acid reactive substance (TBARS) was quantified via the procedure described by Li et al. [23]. Each test was repeated at least three times.

### 2.7. Water Distribution

A low-field nuclear magnetic resonance (LF-NMR) analyzer minispec mq20 (Bruker Optik GmbH, Ettlingen, Germany) was used to analyze water distribution of the mirror carp fillet as the protocol described by Zhong et al. [24]. The mirror carp fillet was cut into a parallelepiped (1 × 1 × 3 cm^3^) and placed in a cylinder NMR tube for measurement. Subsequently, the transverse relaxation time (*T*_2_) was measured using the Carr–Purcell–Meiboom–Gill pulse sequence.

### 2.8. Color

A ZE-6000 colorimeter (Juki Company, Tokyo, Japan) was employed to quantify the CIE parameters of mirror carp fillets. Triplicate measurements of CIE L* (lightness), a* (redness), and b* (yellowness) were obtained from different regions of each fillet to account for potential color variation.

### 2.9. Texture

Mirror carp fillet was cut into a cube (1 × 1 × 1 cm^3^) and the texture (resilience, chewiness, hardness, and springiness) of the cube was assessed using a texture analyzer (Stable Micro System, Godalming, UK) equipped with a P/50 probe. Testing parameters included 2 mm/s pre-test, test, and post-test speed; a 40% compression ratio; and 8 g trigger force.

### 2.10. Statistical Analysis

All measurements were conducted in triplicate, with results expressed as mean ± standard deviation (SD). IBM SPSS Statistics 22.0 was used to perform the data analysis. Differences among the means were evaluated via one-way analysis of variance and followed by a Tukey’s Honestly Significant Difference (HSD) test for post-hoc analyses.

## 3. Results and Discussion

### 3.1. Bactericidal Effect Analysis

**Figure 1:**
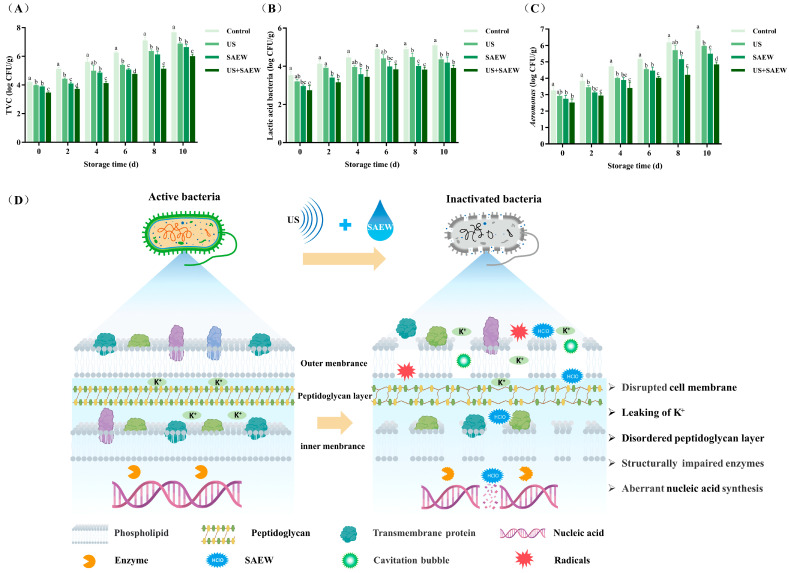
Effect of ultrasonic-assisted slightly acidic electrolyzed water on the counts of total viable (**A**), *Aeromonas* bacteria (**B**), lactic acid bacteria (**C**), and possible disinfection mechanism (**D**) in refrigerated mirror carp fillets. Control: no treatment; US: ultrasonic treatment; SAEW: slightly acidic electrolyzed water treatment; US+SAEW: ultrasonic-assisted slightly acidic electrolyzed water treatment. The means at the same storage time with different letters differ significantly (*p* < 0.05).

Bacterial growth is responsible for the spoilage of aquatic products. During slaughter and gutting, microorganisms from the skin and intestines decompose proteins and lipids, leading to off-flavors and spoilage [25]. TVC serves as a reliable indicator for evaluating the freshness and shelf life of postmortem aquatic products. Generally, freshwater fish with a TVC of 10^6^ CFU/g exceed the edible standard [26]. As shown in Figure 1A, initial TVC in mirror carp fillets treated with the US, SAEW, and US+SAEW decreased by 0.21, 0.30, and 0.73 log CFU/g compared with the control, respectively. Then TVC increased gradually across all groups during refrigeration, though US+SAEW treatment consistently maintained lower counts. After 6 days of refrigerated storage, the microbial load in the control escalated to 6.26 log CFU/g. In contrast, samples treated with either US or SAEW alone reached this microbial threshold (6.00 log CFU/g) until day 8. It was worth noting that the US+SAEW samples remained below this level until day 10, extending shelf life by 4 days. The combined treatment exhibited optimal efficacy, outperforming single US or SAEW application. A similar conclusion was proposed by a previous study by Liu et al. [27], in which US enhanced the bactericidal activity of 77 mg/mL SAEW, and combined treatment reduced the TVC in tuna fillets by 0.86 lg CFU/g. Another study by Suo et al. [28] also reported that the US-assisted SAEW exhibited a stronger bactericidal effect than single treatment and extended the shelf life of Chinese bayberry by an additional 6 days, while single treatment (US or SAEW) extended storage only by an additional 4 days compared to the conventional water washing method.

Our preliminary investigation revealed that *Pseudomonas*, *Aeromonas*, and lactic acid bacteria were the three predominant spoilage microorganisms in refrigerated mirror carp. This observation is consistent with findings by Zhuang et al. [29], who also identified *Pseudomonas* and *Aeromonas* as the primary causative agents of quality deterioration in refrigerated grass carp. The proliferation of lactic acid bacteria in our study might be attributed to the use of zipper bags for fish fillet storage [30]. Our previous investigation specifically evaluated the efficacy of US+SAEW against *Pseudomonas*, demonstrating a 70% reduction in bacterial load [14]. Regarding *Aeromonas* and lactic acid bacteria, as depicted in Figure 1B,C, the US+SAEW treatment significantly improved microbiological quality, achieving the greatest reduction in *Aeromonas* bacteria (0.74 log CFU/g) and lactic acid bacteria (0.79 log CFU/g) counts (*p* < 0.05). Although microbial groups in mirror carp fillets increased progressively during storage, the US+SAEW consistently exhibited the strongest bacteriostatic effect among all treatments. Additionally, research indicated that aquatic products might become contaminated with pathogenic microorganisms such as *Listeria monocytogenes*, *Escherichia coli*, *Staphylococcus aureus,* and *Salmonella* spp. when exposed to contaminated aquatic environments or subjected to inadequate sanitary conditions during processing and transportation, leading to significant deterioration in both freshness and safety parameters [31,32]. Emerging evidence suggested that US+SAEW demonstrated effective inactivation against such pathogens [33,34]. Consequently, this combined treatment showed promising potential for removing diverse microbial populations in fish products. Building on our current findings, we propose that US+SAEW represents a robust intervention strategy for simultaneously preserving fish freshness and ensuring microbial safety.

The disinfection mechanism of US+SAEW is illustrated in Figure 1D. The collapse of cavitation bubbles formed by ultrasound will produce transient heat, high pressure, and shear force, which destroys cell walls and forms micro-cracks in the bacterial cell membranes [7]. Concurrently, the accumulation of large amounts of free radicals can oxidize the lipids on the cell membrane [35]. Such cells will be more accessible to SAEW. SAEW contained abundant HClO, which could act on microbial cells and lead to metal ion leakage and peptidoglycan layer damage [15,36]. Further, after SAEW entered the cytoplasm, the chlorine oxidized sulfhydryl groups of the bacterial enzymes involved in carbohydrate metabolism, which drove the changes in enzyme structure and activity [34]. Moreover, the normal cellular metabolism was disrupted, characterized by nucleic acid fragmentation and impaired biosynthesis [37]. Our prior study has proved that US+SAEW significantly inhibited dehydrogenase and ATPase activities in *Pseudomonas* while concurrently inducing intracellular protein and nucleic acid leakage [14].

### 3.2. pH Analysis

**Figure 2:**
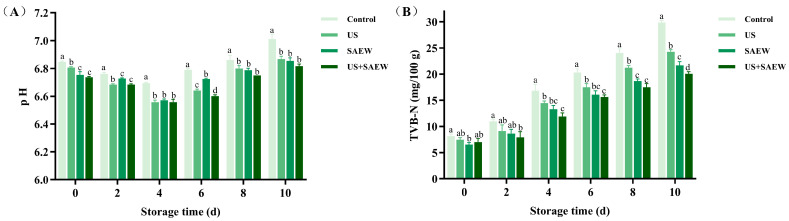
Effect of ultrasonic-assisted slightly acidic electrolyzed water on pH (**A**) and total volatile basic nitrogen (TVB-N) (**B**) in refrigerated mirror carp fillets. Control: no treatment; US: ultrasonic treatment; SAEW: slightly acidic electrolyzed water treatment; US+SAEW: ultrasonic-assisted slightly acidic electrolyzed water treatment. The means at the same storage time with different letters differ significantly (*p* < 0.05).

The pH of aquatic products is related to microbial activity and serves as a critical quality indicator [38]. As shown in Figure 2A, fresh mirror carp fillets exhibited an initial pH of 6.85. During refrigeration, the pH of all samples displayed an initial decline within four days, followed by a gradual increase. This early acidification phase might be attributed to lactic acid production by acidogenic bacteria and muscle glycogen glycolysis [39], while the subsequent pH increase resulted from alkaline nitrogen compounds generated by spoilage bacteria [40]. Notably, samples treated with US, SAEW, or US+SAEW showed significantly lower pH than the control (*p* < 0.05), with the US+SAEW group showing the slowest increase, indicating superior microbial inhibition. Prior limited studies from Lan et al. [20] and Liu et al. [27] have also demonstrated that US+SAEW more effectively stabilized pH compared to individual disinfection technology.

### 3.3. Total Volatile Basic Nitrogen (TVB-N) Analysis

TVB-N denotes the content of volatile basic nitrogen compounds produced by proteolytic bacteria in aquatic products [41], which can be documented as an important indicator for freshness and shelf life [42]. A TVB-N level of 20 mg/100 g of muscle was considered the threshold for edible freshwater fish [43]. As illustrated in Figure 2B, the initial TVB-N of fresh mirror carp samples was 8.17 mg/100 g. Then all groups showed a gradual increase in TVB-N value at different rates with the increase of refrigerated storage time due to the growth of proteolytic bacteria and autolytic enzymes in fish, which break down proteins to generate basic nitrogenous compounds [41]. The TVB-N of the control group exceeded 20 mg/100 g on day 6, while US and SAEW treatments delayed this limit to days 8 and 10, respectively. Notably, the TVB-N of the US+SAEW remained at an acceptable value of 19.10 mg/100 g by day 10, demonstrating superior preservation efficacy. This result suggested that US+SAEW inhibits microbial growth and protein decomposition [42], significantly retarding TVB-N accumulation (*p* < 0.05). Du, Lan, and Xie [44] also reported that a combined pretreatment of SAEW with allicin and antioxidant of bamboo leaves effectively inhibited microbial degradation of protein and non-protein nitrogen compounds in refrigerated bullfrog. This combined intervention consequently suppressed TVB-N accumulation and delayed freshness deterioration.

### 3.4. Protein and Lipid Oxidation Analysis

**Figure 3:**
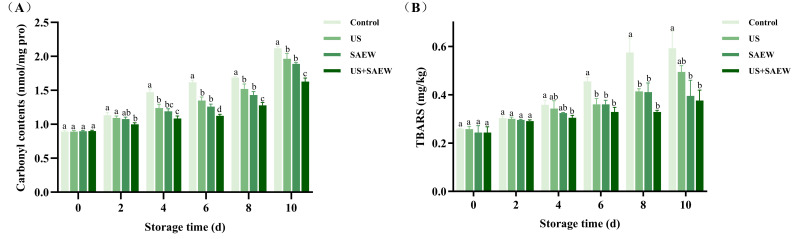
Effect of ultrasonic-assisted slightly acidic electrolyzed water on protein carbonyl content (**A**) and thiobarbituric acid reactive substance (TBARS) (**B**) in refrigerated mirror carp fillets. Control: no treatment; US: ultrasonic treatment; SAEW: slightly acidic electrolyzed water treatment; US+SAEW: ultrasonic-assisted slightly acidic electrolyzed water treatment. The means at the same storage time with different letters differ significantly (*p* < 0.05).

Section 3.3 demonstrated that TVB-N was closely associated with protein oxidation and degradation. Microbial activity induces protein oxidation, leading to conformational loosening and degradation of proteins. This process generates various nitrogen-containing compounds that contribute to TVB-N formation [45]. Therefore, protein oxidation serves as a critical indicator for evaluating the overall quality of aquatic products. Carbonyl compounds, which primarily originate from metal-catalyzed reactions involving basic amino acids (including arginine, lysine, proline, and threonine) in protein side chains, can effectively reflect the protein oxidation degree. Additionally, lipid oxidation products may interact with amino groups to further promote carbonyl formation [46]. As shown in Figure 3A, fresh mirror carp fillets exhibited minimal carbonyl content, and non-thermal disinfection treatments in this study showed negligible effects on the protein oxidation of fresh samples. During subsequent refrigerated storage, all treatment groups displayed progressive accumulation of carbonyl compounds. This accumulation induced protein cross-linking, consequently disrupting the conformational stability of myofibrillar proteins and significantly impairing water retention capacity [41]. This finding was consistent with the LF-NMR results discussed later. Notably, the US+SAEW treatment consistently maintained the lowest protein carbonyl content throughout refrigerated storage, reaching only 1.63 nmol/mg by day 10. In contrast, the control, US, and SAEW showed significantly higher values of 2.12, 1.96, and 1.89 nmol/mg, respectively. These results demonstrated that the US+SAEW treatment effectively prevented protein oxidation of mirror carp fillets during refrigerated storage, exhibiting superior preservation efficacy. Shi, Mei, and Xie [45] demonstrated that arginine, lysine, and proline in fish muscle were readily oxidized into semialdehydes, which account for approximately 70% of total carbonyl compounds. Their research further revealed that ginger essential oil-based active packaging effectively delayed oxidation-induced protein carbonylation of crucian carp during cold storage by suppressing microbial activity.

The lipid oxidation could cause the loss of nutritional value and the production of unpleasant smells of aquatic products, thereby shortening their shelf life [47]. TBARS is a valuable index to evaluate the degree of lipid oxidation. Changes in TBARS of all mirror carp samples were demonstrated in Figure 3B. There were no significant differences in initial TBARS values among these four groups (*p* > 0.05). Existing studies indicated that US treatment might induce lipid oxidation in food through radical generation. For instance, Cheng et al. [9] found that high-intensity US (400 W, 30 min) significantly accelerated lipid oxidation in large yellow croaker. Similarly, Cheng et al. [10] reported that US treatment (250 W, 30–90 min) elevated TBARS levels in Tibetan pork. In contrast, our study employed milder US parameters (200 W, 5 min) combined with SAEW, achieving effective microbial inactivation without triggering significant lipid oxidation. This aligned with Cichoski et al. [17], who observed no detectable lipid oxidation in chicken breast treated with 230 W US for 10 min, as well as Tang et al. [48], who demonstrated that 100 W US combined with plasma-activated water for 30 min simultaneously reduced microbial counts and TBARS in crucian carp on day 0. Collectively, these findings highlighted the critical need to optimize US intensity and duration to balance antimicrobial efficacy with oxidative stability in food preservation applications. The TBARS values of all samples displayed upward trends with the extension of refrigerated time. During this period, the TBARS of the US, SAEW, and US+SAEW were significantly lower than that of the control (*p* < 0.05). The US+SAEW treatment most effectively suppressed lipid oxidation in mirror carp, likely due to its antimicrobial action. Since microbial activity was the primary contributor to lipid oxidation in refrigerated fish, the reduction in bacterial growth by US+SAEW indirectly delayed oxidative degradation during storage [27].

### 3.5. Water Distribution Analysis

**Figure 4:**
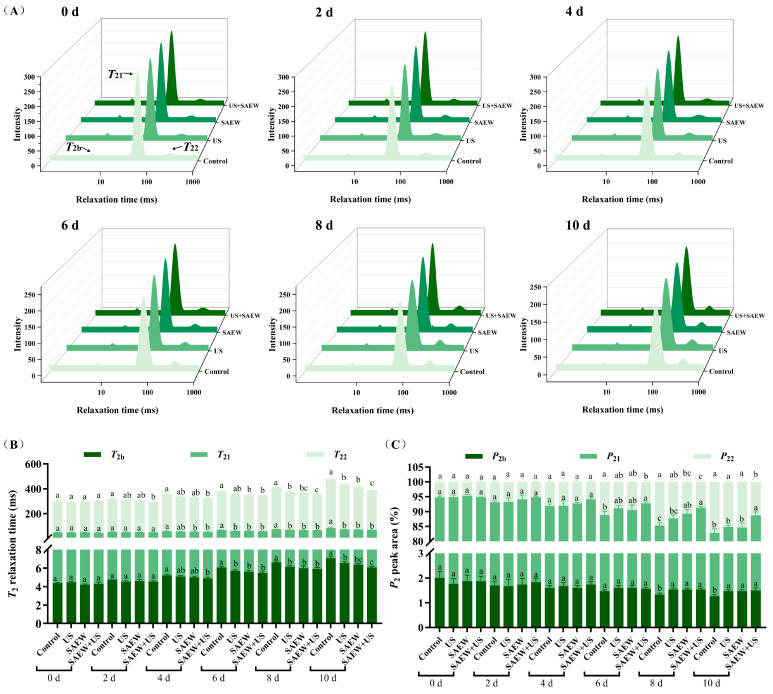
Effect of ultrasonic-assisted slightly acidic electrolyzed water on water distribution (**A**), *T*_2_ relaxation time (**B**) and water peak area (**C**) in refrigerated mirror carp fillets. *T*_2b_: bound water; *T*_21_: immobilized water; *T*_22_: free water; *P*_2b_: percentage of bound water; *P*_21_: percentage of immobilized water; *P*_22_: percentage of free water. Control: no treatment; US: ultrasonic treatment; SAEW: slightly acidic electrolyzed water treatment; US+SAEW: ultrasonic-assisted slightly acidic electrolyzed water treatment. The means at the same storage time with different letters differ significantly (*p* < 0.05).

The water distribution in aquatic products can be used to evaluate the water-holding capacity and quality changes of aquatic products [24]. Three relaxation components were distinguished in Figure 4A: bound water (*T*_2b_, 1–10 ms), immobilized water (*T*_21_, 10–100 ms), and free water (*T*_22_, 100–1000 ms). As summarized in Figure 4B, initial *T*_2_ (*T*_2b_, *T*_21_, and *T*_22_) relaxation times exhibited no differences among treatments (*p* > 0.05), suggesting that US+SAEW treatment preserved the native water-protein binding state, consistent with the findings by Zhao, You, and Wu [49]. During storage, *T*_21_ increased significantly, with partially immobilized water migrating to free water, indicating progressive protein degradation and weakened water-binding capacity due to microbial activity [50]. US+SAEW treatment effectively inhibited the water migration of mirror carp samples. *P*_2_ represents the integral area percentage of *T*_2_ peaks, which reflects the relative content of water confined to the protein structure. As depicted in Figure 4A,C, the initial *P*_2_ (*P*_2b_, *P*_21_, and *P*_22_) of mirror carp samples was not changed by preservation treatment. However, refrigeration progressively decreased *P*_2b_ and *P*_21_ while increasing *P*_22_, indicating that microbial reproduction caused a decrease in the water-binding capacity of mirror carp protein. The US+SAEW combination significantly mitigated these changes by inhibiting microbial proteolysis, thereby maintaining protein-water interactions and overall water retention capacity [51]. Although direct evidence remains scarce regarding how US+SAEW prevented moisture migration by inhibiting myofibrillar protein degradation, a study by Lan et al. has provided a plausible mechanistic analogy. Their study revealed that combined treatment with SAEW and ɛ-polylysine-chitooligosaccharide Maillard reaction products effectively suppressed microbial proliferation and myofibril degradation in sea bass, consequently maintaining high water-holding capacity during refrigerated storage [42].

### 3.6. Color Analysis

**Figure 5:**
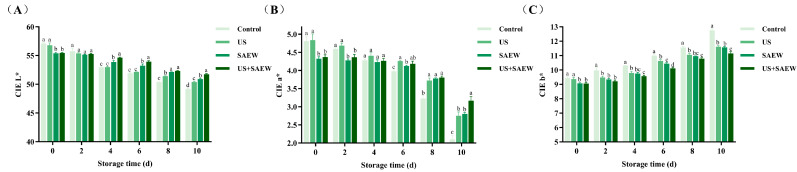
Effect of ultrasonic-assisted slightly acidic electrolyzed water on CIE L* (**A**), a* (**B**) and b* (**C**) in refrigerated mirror carp fillets. Control: no treatment; US: ultrasonic treatment; SAEW: slightly acidic electrolyzed water treatment; US+SAEW: ultrasonic-assisted slightly acidic electrolyzed water treatment. The means at the same storage time with different letters differ significantly (*p* < 0.05).

Color is a direct indicator of freshness and a key factor influencing consumer preference. Figure 5 showed the changes in color values of the mirror carp fillets during storage. Immersion treatments (SAEW and US+SAEW) significantly reduced the initial CIE L* value compared to the control (*p* < 0.05), which was related to the changes in light absorption/scattering properties [5]. The mirror carp fillets in the SAEW and US+SAEW also exhibited lower CIE a* and CIE b* values (*p* < 0.05), potentially from pigment leaching induced by low pH and chlorine in SAEW [25]. During storage, a decrease in CIE L* and CIE a* values and an increase in CIE b* value were observed in all mirror carp fillets. Notably, US+SAEW treatment most effectively inhibited the color changes (*p* < 0.05), with final CIE b* values measuring 12.75 (control), 11.62 (US), 11.58 (SAEW), and 11.15 (US+SAEW), respectively. This preservation effect may result from delayed oxidation of polyunsaturated fatty acids [52], consistent with the lipid oxidation analysis in this study.

### 3.7. Texture Analysis

**Figure 6:**
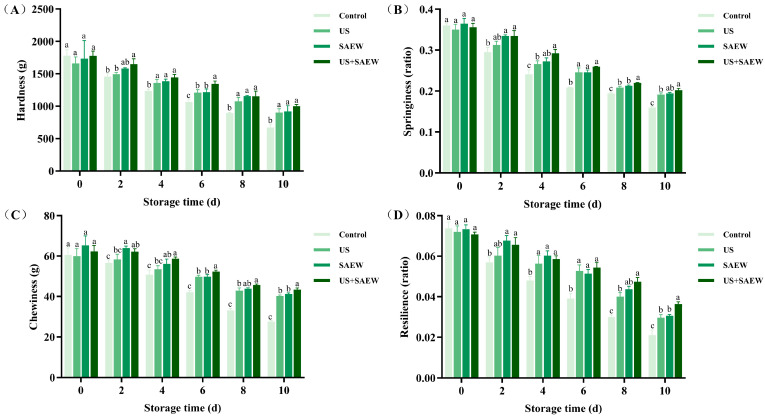
Effect of ultrasonic-assisted slightly acidic electrolyzed water on hardness (**A**), springiness (**B**), chewiness (**C**) and resilience (**D**) in refrigerated mirror carp fillets. Control: no treatment; US: ultrasonic treatment; SAEW: slightly acidic electrolyzed water treatment; US+SAEW: ultrasonic-assisted slightly acidic electrolyzed water treatment. The means at the same storage time with different letters differ significantly (*p* < 0.05).

Texture is a valuable attribute that affects the sensory properties of products, which is affected by the microbiological and autolytic processes [47]. Figure 6 showed the changes in the texture of mirror carp during refrigerated storage. Initial texture profiles (springiness, resilience, chewiness, and hardness) showed no significant differences among treatment groups (US, SAEW, and US+SAEW). However, progressive texture deterioration occurred across all groups during storage, with the US+SAEW demonstrating superior retention. After 10 days, mirror carp fillets in the US+SAEW retained better texture properties, demonstrating only 43.22%, 48.10%, 30.41%, and 43.80% loss in springiness, resilience, chewiness, and hardness, respectively, versus 55.65%, 70.14%, 54.50%, and 62.35% loss in the control, respectively. A gradual textural deterioration was observed in stored aquatic products, presumably caused by microbial proliferation-accelerated protease secretion that mediated myofibrillar protein breakdown and consequent tissue softening [53]. The enhanced texture preservation in combined treatment likely stems from the inhibition effect on bacterial proliferation and enzymatic activity, collectively stabilizing structural proteins [38]. These findings corroborated previous reports on texture preservation in seafood matrices through US+SAEW integration [20] and were consistent with the observed microbial inhibition patterns in this study. Our current research has characterized the color and textural properties of fish fillets, and sensory properties, including odor and taste, can also critically influence consumer acceptance in practical applications. For instance, Li et al. [18] and Yang et al. [19] reported that US-assisted SAEW improved sensory attributes such as appearance, texture, and flavor in onion and sweet potato during storage, demonstrating the potential of this combined technology to enhance the quality and consumer acceptance of vegetables. Current research on the sensory evaluation of US-assisted SAEW for fish freshness retention remains insufficient. Future studies should incorporate standardized sensory evaluation protocols to assess the appearance and flavor of mirror carp fillets during refrigeration and validate the suitability of this combined technology for industrial applications.

## 4. Conclusions

The effect of US-assisted SAEW on refrigerated preservation of freshwater fish fillets was systematically investigated. The US+SAEW treatment demonstrated superior microbial inactivation efficacy, achieving both significant initial bacterial reduction and sustained suppression of microbial proliferation throughout storage compared to the control and individual treatments. The antimicrobial mechanism was associated with inhibited oxidation of protein and lipid, thereby effectively maintaining quality in mirror carp fillets. Notably, this combined treatment extended the shelf life of mirror carp fillets to 10 days, representing a four-day extension compared to the control and at least a two-day improvement over individual treatment. These findings identify US+SAEW as a promising non-thermal preservation technology that combines environmental sustainability with processing effectiveness. For practical implementation, subsequent research should focus on the optimization of processing parameters for industrial-scale applications, an economic feasibility analysis for commercial adoption, and an investigation of potential applications for other aquatic products.

## Data Availability

The original contributions presented in the study are included in the article, further inquiries can be directed to the corresponding author.
